# The Problem of Fairness in Synthetic Healthcare Data

**DOI:** 10.3390/e23091165

**Published:** 2021-09-04

**Authors:** Karan Bhanot, Miao Qi, John S. Erickson, Isabelle Guyon, Kristin P. Bennett

**Affiliations:** 1 Department of Computer Science, Rensselaer Polytechnic Institute, Troy, NY 12180, USA; qim@rpi.edu (M.Q.); bennek@rpi.edu (K.P.B.); 2 OptumLabs, Eden Prairie, MN 55344, USA; 3 Rensselaer Institute for Data Exploration and Applications, Troy, NY 12180, USA; erickj4@rpi.edu; 4 LISN, CNRS/INRIA, Université Paris-Saclay, 91190 Gif-sur-Yvette, France; guyon@chalearn.org; 5 ChaLearn, San Francisco, CA 94115, USA; 6 Department of Mathematics, Rensselaer Polytechnic Institute, Troy, NY 12180, USA

**Keywords:** synthetic data, healthcare, fairness, covariate, temporal, time-series, disparate impact, health inequities

## Abstract

Access to healthcare data such as electronic health records (EHR) is often restricted by laws established to protect patient privacy. These restrictions hinder the reproducibility of existing results based on private healthcare data and also limit new research. Synthetically-generated healthcare data solve this problem by preserving privacy and enabling researchers and policymakers to drive decisions and methods based on realistic data. Healthcare data can include information about multiple in- and out- patient visits of patients, making it a time-series dataset which is often influenced by protected attributes like age, gender, race etc. The COVID-19 pandemic has exacerbated health inequities, with certain subgroups experiencing poorer outcomes and less access to healthcare. To combat these inequities, synthetic data must “fairly” represent diverse minority subgroups such that the conclusions drawn on synthetic data are correct and the results can be generalized to real data. In this article, we develop two fairness metrics for synthetic data, and analyze all subgroups defined by protected attributes to analyze the bias in three published synthetic research datasets. These covariate-level disparity metrics revealed that synthetic data may not be representative at the univariate and multivariate subgroup-levels and thus, fairness should be addressed when developing data generation methods. We discuss the need for measuring fairness in synthetic healthcare data to enable the development of robust machine learning models to create more equitable synthetic healthcare datasets.

## 1. Introduction

The COVID-19 pandemic brought to the forefront the urgent need to rapidly create healthcare solutions and responses to emerging and existing health problems. Data driven approaches based on artificial intelligence (AI), machine learning (ML) and statistics offer powerful ways to rapidly address these problems. Medical data records are generated by millions of individuals everyday, creating an abundance of data to be used for developing healthcare solutions and facilitating new research. For example, electronic medical records (EMRs) can enhance patient care, reduce costs, identify eligible patients easily and improve research efficiency [1]. Healthcare data can streamline clinical research processes and improve data quality [2]. Data in healthcare are also being used for disease progress modeling, risk analysis and much more [3]. Use of supervised and unsupervised machine learning on public health data has been used for outbreak detection, hospital readmission, feature association with outcomes and more [4]. However, irrespective of the abundance of healthcare data, research and work in the field is often restricted due to limited public access to healthcare records. The records are protected by privacy laws like the Health Insurance Portability and Accountability Act (HIPAA) in the United States [5,6] and General Data Protection Regulations (GDPR) in the European Union [7]. Even though these laws allow access to de-identified information, the process of de-identification delays research, is quite costly and can lead to large penalties if data get leaked. In addition to standard de-identification methods, such as binning ages, lose information that may results in loss of the utility of data for some tasks.

Many research articles based on EMR are thus not accompanied by any data, hindering future research and validation. The release of synthetic healthcare data offers a viable solution to provide timely access to EMR data without compromising patient privacy. This would be highly effective during a healthcare crisis such as the current COVID-19 pandemic. Access to COVID-19 data can be extremely fruitful, enabling widespread research and progress. Access to the data can enable exploration of alternative strategies to combat COVID-19 spread [8,9]. Synthetic COVID-19 healthcare datasets can come to our rescue and have been shown to be useful as proxies for real data [10].

The COVID-19 pandemic also revealed great health inequities around the world. In the United States (US), racial and ethnic minorities suffered far greater rates of COVID-19 mortality [11]. Health inequities have been a long-term problem. National Institute of Health (NIH) policies exist to create more representative randomized clinical trials such that clinical trial results include diverse subgroups [12,13]. To support the development of equitable analysis and fair predictive models, it is essential that synthetic versions of healthcare data faithfully represent diverse minority subgroups. Inspired by machine learning fairness research, we refer to this as the “synthetic data fairness problem”.

**Figure 1:**
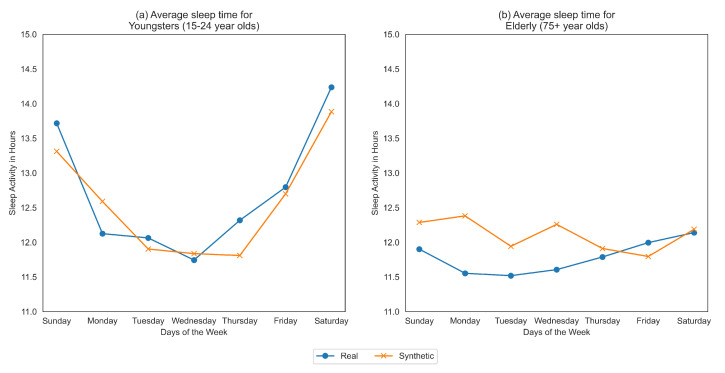
Covariate plots for average sleep times across the week for real (blue) and synthetic (orange) American Time Use Survey dataset: (**a**) 15–24 year old and (**b**) 75+ year old. Sleep habits for individuals aged 15–24 and Elderly people aged 75 years differ markedly. Synthetic data more accurately represents the former than the later.

Health inequities among different populations is an ongoing health problem. Inequitable representation of subgroups in synthetic data could lead to inaccurate analysis where conclusions and predictive models do not generalize to the real data. Commonly in EMR, health outcomes are influenced by covariates such as age, gender, race, obesity and comorbidities. In ML fairness, these covariates are often referred to as “protected attributes”. To be “fair”, a synthetic data model must ensure that the synthetic data represent the real data across different subgroups defined by these covariates/protected attributes. For example, in the American Time Usage Study (ATUS) [14], the average sleep duration of an individual is affected by their age, thus the synthetic data must correctly capture the proportion of subjects in each age group and accurately represent the sleep habits within each subgroup. Figure 1 demonstrates that sleep habits of elderly people (aged 75+ years old) are more poorly represented than the subgroup of aged 15-24 year olds using “co-variate plots” based on the synthetic data generated in [15].

Synthetic healthcare data can be useful when (i) it ensures patient privacy, (ii) it has high utility (e.g., produces high quality ML models), and simultaneously (iii) it has good resemblance to the real data from which it is derived [16]. While this has been shown to be effective at the general level, only limited exploration has been done with covariates. In our recent work, we discuss the covariate analysis for synthetic data evaluation using time-series metrics to highlight the importance of covariate-level resemblance [17]. This begs the question, is a given synthetic generation method “fair” towards real data subgroups or does it discriminate against certain protected classes to achieve the balance between these aspects of synthetic data generation? We assert that, while utility, resemblance and privacy are key to synthetic data generation, they must be accompanied by (iv) fairness measures relevant to each.

Resemblance in synthetic data generation is a measure of how closely matched are real data (used for training the synthetic data generator) and synthetic data generated by the model. One might argue that we should make the synthetic data as closely resembling to the available real data, but this may yield overfitting and violate the requirement that privacy be preserved by regurgitating examples that too closely resemble the actual subjects. Rather, one should target the resemblance between the synthetic data distribution and the underlying real data distribution, not the exact training samples drawn. Thus, there is a trade-off between resemblance (between synthetic data and real training data) and privacy, similar to the fit vs. robustness trade-off. Alas, avoiding overfitting is only a necessary condition to obtain privacy-preserving data [18], additional steps must be taken to downgrade the data to protect privacy, which in turn can effect utility [19].

In this article, when measuring resemblance, our goal is to ensure that the real and synthetic data distributions match at similar levels for all protected subgroups. A synthetic data generation method must capture the intrinsic trends and patterns in the real data, while simultaneously preserving privacy of subjects in all subgroups. To achieve this resemblance, we want to ensure that the synthetically generated data do not deviate significantly from the real data such that it discriminates against certain protected subgroups.

This requires the application of novel fairness metrics that measure equity in synthetic data resemblance. We propose measuring this fairness in resemblance using representational rates and time-series resemblance analysis at the covariate-level. It is important to highlight the distinction between the definitions of “resemblance” and “representation” in the context of this work. “Resemblance”, as mentioned above, is how close is the synthetic data are from the real data while “representation” is used in the context of fairness where based on the fairness metric, it highlights how over, adequate or under-represented a given subgroup is.

Fairness should be part of each aspect of synthetic health data and should be evaluated for the generation method. We propose that fairness in synthetic health data should be measured at each evaluation step. Thus, in this article, we propose metrics for “fairness in resemblance” of the synthetic data with the real data. We also briefly discuss about “fairness in utility” and “fairness in privacy” but studying such aspects has been left as a potential future work.

We demonstrate potential problems with synthetic data fairness using three different synthetic healthcare datasets in prior published research studies [15,16,20]:

Case Study 1 examines the fairness of synthetic version of an extract from the popular MIMIC-III dataset designed to duplicate a prior research study [16]. The research study explored the impact of race on 30-day mortality based on odds-ratio of various comorbidities and demographic information [21]. We adapt a metric (log disparity) and a statistical test developed to evaluate the representativeness of the subgroups in randomized clinical trials to find inequities in the synthetic data as compared to the original data. We examine the superset of subgroups defined with respect to the protected attributes, and find subgroups with significant differences in the *proportion* of subjects in subgroups occurring in the original and synthetic data.

Case Study 2 explores the fairness of synthetic sleep data generated for various age-groups and genders on the American Time Use Survey (ATUS) dataset [15]. Examining all subgroups using the log disparity metric demonstrates that the synthetic data capture the proportion of subgroups quite well. Previously authors used covariate plots to qualitative compare the means of the real and synthetic sleep data for different subgroups (similar to Figure 1). These inferences are subject to viewer bias and expertise, leading to inconsistent results. Thus, we propose new metrics to evaluate fairness in subgroups across temporal trends which build on prior time-series resemblance metrics for synthetic data [17]. We propose the synthetic data fairness measurement using modified time-series log disparity metric. Here, we apply two different time-series metrics, Pearson’s Correlation Coefficient (PCC) and Directional Symmetry (DS) to demonstrate the problem of fairness across the temporal trends of covariate subgroups. The results showed that the synthetic data did not capture the trends well especially for older people (age 75 and above), indicating bias in synthetic data.

Case Study 3 examines synthetic data from a study on Autism Spectrum Disorder (ASD) claims data [20] by applying the time-series log disparity metric. These data significantly deviate from the other two, given that it is multivariate (having seven distinct time-series corresponding to different diagnoses) and being exceptionally big (280 K+ records). The results demonstrated that the synthetic dataset has significant biases in the representation of females and some racial/ethnic minority subjects for some diagnoses time-series.

Fairness analysis of synthetically generated healthcare data is essential to preserve utility of synthetic data used in place of real medical data. Covariate-level analysis of fairness metrics provides a comprehensive understanding of this generated synthetic data, enabling us to explain its utility and resemblance better. This article exposes the bias that exists in published synthetic datasets towards certain protected attributes through three case-studies of healthcare datasets. Admittedly, the problem of fairness is quite broad with many existing definitions of fairness [22] underscored by the condition that not all can be satisfied at the same time [23]. We therefore present two ways to measure synthetic healthcare fairness, while acknowledging the limitation that these are not comprehensive metrics catering to all definitions of fairness nor do they comprehensively capture the many measures of similarity of real and synthetic data. We propose this article as a starting point for future research in this domain, potentially developing more robust metrics for synthetic healthcare data fairness evaluation and making them a routine part of synthetic data evaluation.

The results in this article, have the following structure:

- We introduce metrics for synthetic healthcare data fairness evaluation for covariate-level insights.
- We then highlight the inequities in published synthetically generated datasets using three case-studies.
- We demonstrate the need for analysis of covariate-level fairness in synthetically generated healthcare data.
- Finally, we discuss some future research directions towards making synthetic generators more equitable.

We follow with discussion and details of the methods employed.

### 1.1. Related Work

Synthetic data generation involves the creation of realistic looking data using a specific method. In the healthcare domain, synthetic data generation requires that the privacy of the patients in the real data is always maintained while achieving high level of utility and resemblance. Generative Adversarial Networks (GANs) [24] like medGAN [25], HealthGAN [20] etc. provide promising solutions that do not require real health data once they are fully trained. As HealthGAN promises the juxtaposition of utility, resemblance and fairness with recently published work (catering to categorical, binary and temporal data) [20], we focus on the analysis of several available synthetic datasets produced by HealthGAN for this work.

Machine learning models based on biased data have been shown to discriminate based on gender, race etc. [26]. Very recently, synthetic data generation methods have begun to adopt fairness as one of the key elements of equitable data generation. Differentially private GANs attempt to preserve privacy and mitigate fairness but have shown to lead to decreased synthetic image quality and utility [27]. Others measured synthetic data fairness by developing models on the data and then calculated fairness metrics like demographic parity, equality of odds etc. [28]. They too found that models for differentially private data result in more disparity and poorer fairness results. In [27], the authors looked at group fairness metrics like parity gap, specificity gap, recall gap, etc. on a downstream model generated on the synthetic data. Authors of Fairness GAN also used error rate, demographic parity and equality of opportunity as fairness metrics [29]. Equalized Odds is another metric used for differential privacy learning fairness [30]. However, very limited research has been done on fairness of subgroups of covariates, opening a potential area of further exploration of synthetic health data generation.

### 1.2. Metrics for Health Data Equity

To evaluate healthcare data inequity, researchers developed metrics to compare the observed enrollment rates of protected subgroups with a reference group (i.e., enrollment fractions [31]) or measure the proportion of target population eligible for the trials (i.e., GIST [32,33]). Our recent work developed theorems to create scalable Randomized Clinical Trials (RCT) versions of ML metrics that quantify equities of protected subgroups with respect to any community or population level targets [34].

The definition of the term “fairness” has a number of definitions that exist in ML literature with corresponding metrics. As per the impossibility theorem of fairness [23], not all the metrics can be satisfied at the same time, each having their own specific trade-offs.

In synthetic health data generation, we define “fairness” as the ability of the generation method to capture the distribution of the real data subgroups effectively across all protected attributes. Put simply, a fair synthetic health data generation method would produce similar subgroup statistics and trends, leading to real and synthetic data distributions that match at similar levels for all protected subgroups. A synthetic data set could be unfair even if quality metrics applied to the entire synthetic data set are very good. For example, the synthetic data could represent the distribution of a very large majority subgroup very well, but fail to accurately represent the distribution of a small protected subgroup.

Thus, an “equity metric” should measure unwanted bias in the synthetic data when compared to the real data. Here, we define the equity metric in terms of “disparate impact” which measures the bias in two different groups receiving significantly different outcomes as it has been the focus of many recent publications [35,36,37,38]. For Randomized Clinical Trials (RCTs), we previously designed a set of metrics [34] to measure RCT representativeness using representational rates based on ML fairness metrics [39].

In ML, disparate impact is used to quantify the fairness of a trained classification function. Let each data point $x\in {ℜ}^{d}$ have an associated label y∈{0,1} which also has a predicted label $y^{\prime }\in \left\{0,1\right\}$ produced by the classification function. We assume y=1 is the desired positive outcome, similar to previous works. Protected attributes are defined as any subset of the *d* attributes that can discriminate the population into different groups in which parity is desired in terms of outcomes. A protected subgroup is defined based on one or more protected attributes by a binary membership function g(x). If g(x)=1, then the subject *x* is in the protected subgroup, otherwise if g(x)=0, it is not. For ease of notation, g(x) can be appropriately redefined to represent any particular subgroup.

The ML fairness disparate impact measures the positive prediction rate of both the protected and unprotected groups, requiring them to be similar under the “80 percent rule” defined by the US Equal Employment Opportunity Commission (EEOC) [40]. Under this rule, the positive outcome prediction of a given subgroup should be at least 80% of the other subgroups to achieve fairness, i.e.,

(1) $$\frac{P(y^{\prime }=1|g\left(x\right)=1)}{P(y^{\prime }=1|g\left(x\right)=0)}\geq 0.8.$$

These probabilities are estimated by frequencies evaluated on training data and the prediction of the ML classifier on the training data.

Our proposed approach builds based on the log disparity metric for quantifying representativeness of Randomized Clinical Trials (RCTs) developed by Qi et al. [34]. It assumes that there is a theoretical sample of subjects *R*, drawn equitably from a target population, and there is the observed sample of subjects, *S*, that were actually recruited in the RCT. The observed sample may or may not be equitable. According to a general mapping from ML fairness to RCTs, the ML disparate impact metric is transformed into its RCT version, log disparity, to measure how representative the subgroup $g\left(x\right)=1$ in the actual clinical trial sample *S* was as compared to target population sample *R*. The log disparity metric for RCT is

(2) $$\log\left(\frac{\mathrm{odds}(g(x)=1|x\in S)}{\mathrm{odds}(g(x)=1|x\in R)}\right).$$

where odds(g(x)=1|x∈S)=P(g(x)=1|x∈S)/(1−P(g(x)=1|x∈S)) and can similarly be defined for odds(g(x)=1|x∈R). For RCTs, P(g(x)=1|x∈S) is estimated from the clinical trial data and P(g(x)=1|x∈R) is estimated from surveillance datasets or electronic medical records.

Log disparity was calculated for all possible subgroups defined by protected attributes and the statistical significance was calculated using two-proportion z-test with the Benjamini–Hochberg correction multiple hypothesis testing. An interactive visualization app based on log disparity helps users efficiently assess representations of all subgroups defined over multiple attributes and to visualize any potential inequities for under-representation or missing subgroups. The colored tables and sunburst charts, similar to those in Figure 3, effectively enable users to investigate multiple subgroups with hierarchical information simultaneously and capture the influence of additional attributes on the existing ones.

## 2. Results

We develop two metrics to quantify fairness on three previously published datasets for MIMIC-III, American Time Use Survey (ATUS) and Autism Spectral Disorder (ASD) claims data for different protected attributes such as age, gender, and race.

We developed a log disparate impact equity metric which is defined as the ratio of odds of subjects of the protected group in the synthetic data to the real data. This is accompanied by statistical tests to identify if the results are statistically significant or not. For temporal healthcare datasets, we present the time-series specific log disparity metric. The metric calculates the resemblance between the real and synthetic time-series for subgroups using two time-series metrics, Pearson’s Correlation Coefficient (PCC) and Directional Symmetry (DS).

The synthetic data try to match the distribution of the real data to achieve utility and resemblance. However, even though it might be able to achieve that on the dataset overall, we are interested to know the resemblance and fairness at the subgroup-level. The metrics are designed to quantify the fairness for subgroups of the protected attributes. These are quantified for both non-temporal and temporal healthcare datasets. The log disparity metric aims to compare the proportions of the subgroups in the synthetic data to the real data, to compute whether the proportions are similar or not. The time-series based disparity metric, quantifies the resemblance in the time-series trends for the synthetic data with the real data. The two metrics enable an outlook on fairness in resemblance for healthcare datasets.

### 2.1. Log Disparity Metric for Fairness of Rates

The definition of log disparity for clinical trials can be extended to synthetic healthcare data generation. Protected attributes are essential for healthcare data research as they reveal the impact of certain attributes on results of a healthcare study. For example, to verify that a certain drug works for both men and women, the research study must incorporate both men and women during their trials. Thus, when real world healthcare data are collected, it includes a certain proportion of individuals having different protected attributes. Thus, if that data need to be used for clinical research, similar proportions must also be represented in the synthetic health data. However, as synthetic data is not exactly the same as real data (ensuring privacy preservation), we cannot directly measure the same subject’s likelihood of being in both the real and synthetic data.

For synthetic data generation, we are concerned with the overall protected attribute representation between the real and synthetic datasets. The observed rate of records with protected subgroup g(x)=1 in the synthetic data *S* should be similar to the observed rate for the same subgroup g(x)=1 in the real data *R*.

(3) P(g(x)=1|x∈S)≈P(g(x)=1|x∈R).

Using the methods created for RCTs [34], the synthetic data fairness metric is derived from the ML-Fairness disparate impact metric Equation (8) and is defined below.

(4) $$\log\left(\frac{\mathrm{odds}(g(x)=1|x\in S)}{\mathrm{odds}(g(x)=1|x\in R)}\right)\;.$$

The membership function g(x) is a binary definition of subgroup, i.e., subject is in subgroup or not based on the selected attributes. In Equation (4), the numerator corresponds to the sample of subjects found in the synthetic data and the denominator represents the sample of subjects found in the real data.

We then adopt the methods developed for visualizations and statistical tests of representativeness of RCTs to synthetic data fairness. As we take the log value for this ratio, we would modify the “80 percent rule” to accommodate this change.

**Figure 2:**
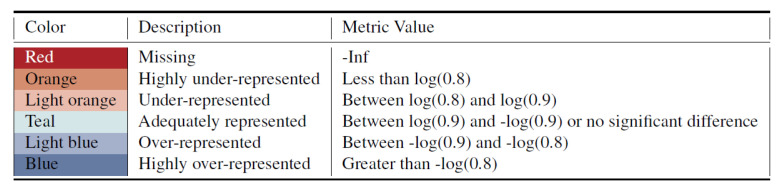
Color codes of 6 representativeness levels based on metric value.

For the proposed metrics, the unfair metric values are further divided by two user-selected thresholds into highly inequitable representation and inequitable representation. We apply the threshold value ±log(0.9) (i.e.,“90 percent rule”) to split the adequately represented values with under-/over-representation and use ±log(0.8) to discriminate more severe unfairness. Therefore, we categorize the metrics values into six levels and represent them as different colors as shown in Figure 2. Red is used to indicate if the subgroup is missing entirely from the synthetic data.

Note that these categories are slightly more restrictive than those previously proposed for log disparity of RCTs [34]. The longitudinal nature of healthcare data is highly important as future poor health events may be influenced by previous events. Thus, it is essential to have stricter bounds for this metric, so we can ensure that the synthetic data fairly captures current real data trends, ensuring better future results.

### 2.2. Time-Series Disparity

Healthcare data are often longitudinal and hence, the synthetic data generation method must fairly capture the time-series trends across various subgroups of protected attributes. To accomplish this, we propose the time-series log disparity metric as a measure of empirically quantifying fairness in real and synthetic data temporal resemblance. The metric can be used to compare covariate time-series for various subgroups of protected attributes in the dataset.

Let us consider a univariate time-series healthcare dataset which consists of protected attributes $x^{p}\in {ℜ}^{d}$, unprotected attributes $x^{u}\in {ℜ}^{d}$ and temporal attributes $x^{t}\in {ℜ}^{m}$. We can stratify this data based on any protected attribute. Let us consider we have a subgroup defined by g(x)=1 and the remainder is defined by g(x)=0. We can merge each corresponding temporal feature for a subgroup using a function *f* to create a univariate time-series for that subgroup. This time series could be from the real data or a sample of the real data, *R*, or from the synthetic data, *S*. The function *f* can be defined using any statistic, for example, the average value, the counts, etc. Thus, the resultant for the time-series $TS_{r}\left(g\left(x\right)=1\right)$ for real data *R* is defined below.

(5) $$TS_{r}={\left\{f(x_{r}^{k})\right\}}_{k=1}^{T}\;\mathrm{for}\;x\in R.$$

Similarly, the same steps can be applied on the synthetic health data *S*. This will result in a time-series for the same subgroup in the synthetic data $TS_{s}\left(g\left(x\right)=1\right)$ defined by Equation (6).

(6) $$TS_{s}={\left\{f(x_{s}^{k})\right\}}_{k=1}^{T}\;\mathrm{for}\;x\in S.$$

For the real and synthetic time-series above, we can calculate a time-series resemblance metric res(). This can be contrasted with a similar resemblance on time-series for the real and synthetic data where g(x)=0. Then we define the time-series log disparity as follows.

(7) $$\log\left(\frac{E(res\left(TS_{r},TS_{s}\right)|g\left(x\right)=1)}{E(res\left(TS_{r},TS_{s}\right)|g\left(x\right)=0)}\right).$$

If the data of interest has only one temporal feature then there are only *T* observations for the time-series. In the case of multiple temporal features, the first *T* observations in the times-series are for the first feature, the next *T* observations are for the next feature, and so on. If a given dataset is not in the above format, it can easily be mapped to this format using the workflow described by Dash et al. [15]. We leverage Pearson’s Correlation Coefficient (PCC) and Directional Symmetry (DS) as two time-series metrics in this article. We note that these are just two examples of time series metrics that could be used. Any other time-series resemblance or error metric could be adapted into an equity metric by stratifying by covariates.

If the dataset is not temporal in nature, the temporal variables $x^{t}\in {ℜ}^{m}$ would not exist for the particular dataset. In that case, covariate-level univariate analysis based on $E(res\left(TS_{r},TS_{s}\right))$ could be calculated for covariate subgroups using any other of the many existing methods for quantifying differences between distributions.

### 2.3. Case Study 1: Impact of Race on 30-Day Mortality Using MIMIC-III Dataset

Multiparameter Intelligent Monitoring in Intensive Care (MIMIC) is a public dataset that includes de-identified information about patient demographics and ICU stays for patients [41]. In [21], the authors used MIMIC-II to identify the impact of Race on 30-day mortality of the patients. A synthetic version of the analyzed dataset was created based on MIMIC-III using HealthGAN and the results were duplicated [16]. Thus, we use the synthetic and real versions of the MIMIC-III dataset for our analysis here.

**Figure 3:**
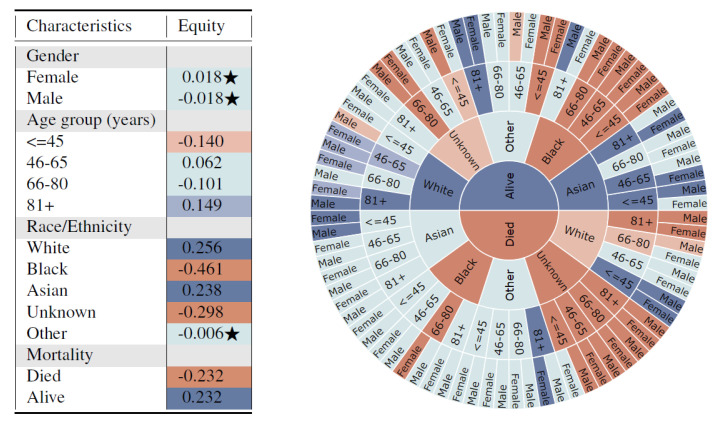
Representativeness results measured by log disparity with protected attributes gender, age, race/ethnicity, and mortality for MIMIC dataset. (**left**) Representativeness of subgroups defined by a single protected attribute. Star indicates that there was no statistically significant difference between subgroups from the real and synthetic datasets. (**right**) Representativeness of multivariate subgroups in sunburst plot with inner to outer rings defined by mortality, race/ethnicity, age and gender, respectively.

The univariate results of the 13 subgroups in Figure 3 (left) shows that unfair synthetic data is generated for different race subgroups when compared with the real data, which has the potential to exacerbate biases towards traditionally under-served groups such as Blacks. Additionally, subjects who died are highly under-represented in the synthetic data.

This captures potential dependencies of variables on a multivariate distribution in a potentially biased data generation model. Furthermore, the sunburst figure clearly indicates a fairness violation for subjects with mortality = death, race/ethnicity = Black, age = 66–80, and gender = Female. This indicates that the synthetic generation model may be severely biased towards certain subgroups and subsequent analysis and decisions based on the synthetic data may not be fair. The proposed method navigates researchers to find places of bias, and helps them model the synthetic data generation more fairly and accurately. For example, additional parity fairness constraints regarding violated protected attributes could be incorporated into the data generation model as is common practice in ML-Fairness research. Furthermore, performance variations across different multi-variate subgroups could be used to evaluate, tune, and improve the generation model.

### 2.4. Case Study 2: Average Sleep Time of Americans based on ATUS Dataset

We used the ATUS dataset to look at the average sleep times of various subgroups [15] using the disparate impact metric. As the dataset is temporal, we also applied the time-series log disparity metric.

**Figure 4:**
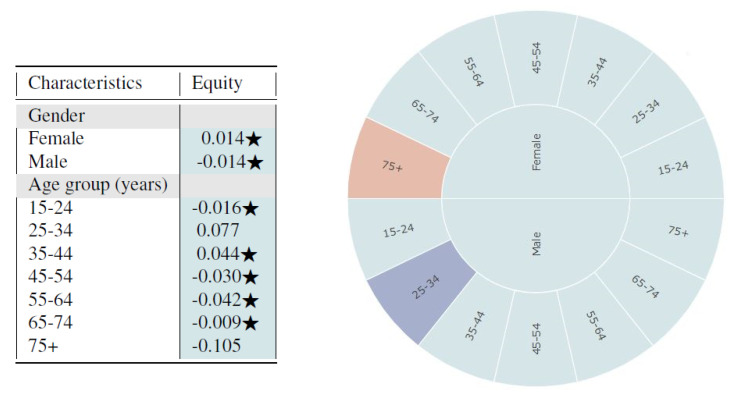
Representativeness results measured by log disparity with protected attributes gender and age for ATUS dataset. (**left**) Representativeness of subgroups defined by a single protected attribute. Stars indicates that there was no statistically significant difference between subgroups from the real and synthetic datasets. (**right**) Representativeness of multivariate subgroups in sunburst plot with inner to outer rings defined by gender and age, respectively.

From Figure 4, we observe that the synthetic data seem to fairly represent all subgroups of gender and age based on the log disparity metric. We find that for most cases, the differences are not even statistically different.

Based on the sunburst, we see only minor multivariate unfairness. Females aged 75 and above are under-represented while males aged 25–34 are over-represented. From observing the figures, one could infer that the synthetic data sample is quite fair in its representation of the proportion of subgroups. However, the metrics based on representational rates are not sufficient for evaluating time-series healthcare datasets since temporal variables are not captured. The multivariate analysis detects some age-relevant unfairness for different gender groups but fails to capture time-series resemblance. Thus, we also applied the time-series log disparity metric on this dataset.

The healthcare data are stratified based on the protected attributes (age, gender) and the resultant time-series for real and synthetic datasets are compared using time-series metrics like Pearson’s Correlation Coefficient (PCC) and Directional Symmetry (DS). The age in the dataset is split into bins: 15–24, 25–34, 35–44, 45–54, 55–64, 65–74 and 75+. We average the sleep time for the various age groups with the results for youngsters (15–24) and elderly (75+) shown in Figure 1.

From Figure 1, we clearly see that the trends themselves between the two extreme subgroups are very varied. Youngsters tend to sleep more during the weekends than weekdays while elder people sleep approximately the same across all days of the week. The change in average time slept throughout the day for youngsters is very high, ranging from 11.5 h to 14.5 h which is in complete contrast to elderly people who sleep somewhere between 11.5 and 12.5 h. We also see that the synthetic data more accurately represents the younger population than the older one.

**Figure 5:**
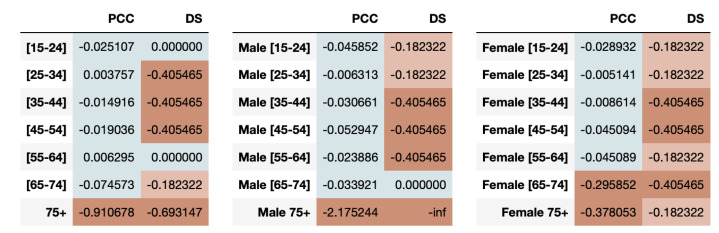
The time-series log disparity metric results for various combinations of protected attributes. People aged 75 or older are always highly under-represented across both genders.

For both youngsters and elderly, even though the average sleep times across days are captured closely by the synthetic data, the directions of the series is often a mismatch between real and synthetic data. This is confirmed by the evaluation of fairness metrics on all the age-groups as shown in Figure 5. We observe that the resemblance in synthetic time-series for youngsters is high, indicating fairness using both PCC and DS metrics. However, the elder population is highly under-represented as determined by both metrics.

We can also measure the fairness of the synthetic data based on combination of age-groups with gender in the dataset. The results of the PCC and DS disparate impact metrics are presented in Figure 5.

We note that males aged 35 to 64 are highly under-represented in capturing the directional trends while females aged 35–54 and 65–74 are highly under-represented. The high under-representation of elders aged 75 or older in the synthetic data is observed in both males and females using PCC disparity. However, when we look at the DS metric results, we notice that the synthetic data perform poorly across almost all combinations of age and genders, hinting at its inability to capture the directional trends for protected attributes in this dataset.

It is interesting to note that the results from the two metrics can be contrasting in some cases. For example, for age-group 15–24 and 25–34, PCC shows the groups to be adequately represented but DS shows the age-group 25–34 being under-represented. This underscores the importance of using a comprehensive set of time-series metrics for gaining better insights into synthetic healthcare data fairness.

### 2.5. Case Study 3: Co-occurring Comorbidities in ASD Patients’ Claims Dataset

The ASD claims data include records of children having multiple diagnosis across a 5-year period used in [42] with synthetic data generated using HealthGAN [20]. The real data were accessed inside a secure environment provided from OptumLabs^®^ Data Warehouse (OLDW), which provides de-identified medical data access. Following OLDW procedures, the HealthGAN synthetic model and summary statistics necessary to calculate the time-series log disparity metric for each time-series/subgroup combination were calculated and exported from OLDW, and then access was terminated. Patient privacy was preserved, since no other additional access to subject data was required.

The real dataset consists of bi-yearly analysis of seven different Comorbid Medical Conditions (CMCs) for each subject. The ASD Prevalence dataset includes records of prevalence of diagnosis for each patient. Each temporal column represents whether the child was diagnosed with that particular CMC during that 6-month period or not. Put simply, each binary column represents whether the child was diagnosed for that disorder (1) or not (0) in that specific time-period. This, creates a time-series of presence or absence of diagnosis across a 10 point time-series for each diagnosis. We calculate the average values of occurrence of the seven CMCs across the 5-year period for each subgroup enabling us to use PCC and DS based time-series log disparity metric for fairness. This creates a set of seven time-series for each subgroup, making it a multivariate time-series problem. Each time-series in the real data is then compared with the corresponding time-series in the synthetic data using the time-series fairness metrics.

The time-series of ASD prevalence values for each gender and ethnicity are compared between the real and synthetic health datasets using the time-series log disparity metric applied to each CMC.

**Figure 6:**
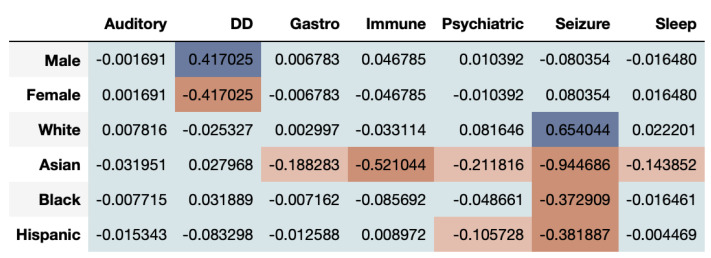
ASD Binary Data: Time-series Log Disparity using the PCC resemblance metric for various subgroups for different CMCs. Males and Whites are well represented (teal) while significant under-representation occurs for other gender and race/ethnicity subgroups for some CMCs.

**Figure 7:**
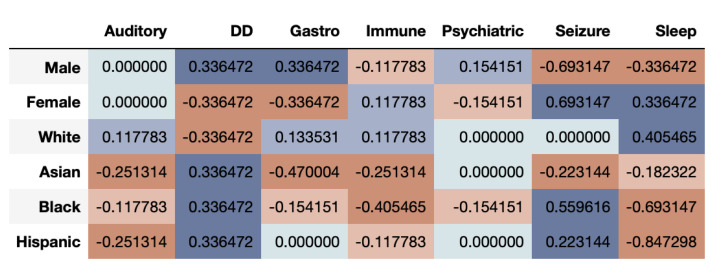
ASD Binary Data: Time-series Log Disparity using the DS resemblance metric for various subgroups for different CMCs. Whites are often over-represented while other groups are under or over-represented depending on the time-series being observed.

Figure 6 shows that the correlational fairness between real and synthetic dataset shows signs of bias. As we can see, the synthetic data under-represents females for DD. Further, Asians are highly under-represented across CMCs including Gastro, Immune, Psychiatric, Seizure and Sleep. The results based on the DS metric as seen in Figure 7, provide a very interesting twist to fairness evaluation. We note that a given subgroup can be over-represented across some time-series while being under-represented across others. Whites seem to be generally well represented or even over-represented across both metrics. This underscores that for while capturing the prevalence of diagnosis in the dataset, the synthetic data over-represent some and under-represent other subgroups. If the exact synthetic dataset is used for any analysis, the resultant may be altered by bias introduced in the synthetic data.

From the results of the ASD data, we can clearly see that multivariate time-series have a more complex structure and make fair synthetic data generation difficult. While some groups are over-represented across a particular time-series, the same groups may be under-represented in others.

## 3. Discussion

The “Synthetic Data Fairness Problem” entails the presence, identification, and ultimately removal of bias and unfairness in synthetically generated data against subgroups defined by protected attributes. The first step in addressing inequity in synthetic data generation is measuring it. In this article, we examined “fairness of resemblance". Resemblance in synthetic data generation is a measure of how closely matched are *real data* and *synthetic data* generated by the model. To examine fairness, we define two metrics based on the resemblance for different subgroups: log disparity which measures the difference of prevalence of protected subgroups and time series log disparity which reveal the relative resemblance across time of subjects. We applied these metrics to real and synthetic data created by HealthGAN from prior publications.

### 3.1. Problems in Published Synthetic Datasets

We demonstrated the presence of unfairness in synthetically generated healthcare datasets using three case studies:

Impact of Race on 30-day Mortality Using MIMIC-III Dataset: Using a very popular medical dataset, MIMIC-III, we found that the synthetic version of the dataset introduced bias across different covariates as identified using the log disparity metric. Whites and Asians were highly over-represented in the synthetic data while Blacks were highly under-represented. On stratifying the results based on grouped covariates, we found that Asians were over-represented for all age-groups except 66–80 for the alive subset. At the same time, Blacks aged up to 80 years were all highly under-represented. The results highlight that different races are being unfairly represented in the synthetic data. MIMIC’s popularity in the healthcare research domain makes this insight quite alarming, where someone might use a synthetically generated dataset without realising the hidden bias.

Average Sleep Time of Americans based on ATUS Data: On observing the sleep times using the log disparity metric, the synthetic dataset appears to adequately represent all genders and ages with a slight over-representation for males aged 25–34 and under-representation of females aged 75 or older. However, as the data are temporal in nature, we evaluated the fairness using the time-series log disparity metric and the results were very biased. Using the PCC metric, both males and females aged 75 or older were highly under-represented. Based on DS metric results, we found that males were under-represented overall when compared to females. The results indicate that looking only at univariate metrics of resemblance and fairness may obscure inequities that would only be revealed when examining multi-variate temporal relations, rendering any future models with high risk of bias.

Co-occurring Comorbidities in ASD Patients’ Claims Dataset: Using time-series log disparity metric on ASD dataset, we found that multivariate time-series are highly complex and synthetic data struggle to capture all time-series trends. These point out areas of improvement for future synthetic data generation methods. We found that Whites are clearly over-represented while Asians are often under-represented. The representation between the two genders vary based on the time-series being considered.

From the metric results of the three case studies across multiple protected attributes, we observe that synthetic data often struggles to capture the proportions that exist in the real data. This can clearly be seen through both log disparity and time-series based metrics. In synthetic healthcare data generation, the data generation method attempts to achieve high utility, and high resemblance while preserving patient privacy. To maintain this privacy, the methods often introduce controlled noise to the real data such that the records generated are realist without being real. However, this is likely to affect the resultant data, leading to different levels of proportions for the various subgroups. This potentially leads to the introduction of bias and fairness in the synthetic data, causing it to deviate from the real data to maintain the trade-offs between utility, resemblance and privacy.

Furthermore, during synthetic data generation, the method does not specifically know what are the protected and unprotected attributes in the dataset, and considers them the same. Thus, it simply tries to replicate the distributions of the real data for all features. As the synthetic data generation process does not consider fairness of the data across protected attributes, it is not bound to exactly replicate the real data. It does not specifically target for equal representation for subgroups of any given protected attribute, potentially leading to changed representations in the synthetic data, and introducing bias.

However, these are problems which can be addressed using generative models which also take fairness into consideration. This shall involve the addition of fairness as one other metric during synthetic data generation such that the model also aims to achieve fairness along with utility, resemblance and privacy.

### 3.2. Need for Covariate Fairness Metrics

We introduced two metrics for quantifying the fairness in synthetic data, for non-temporal and temporal healthcare records.

Metric on Representation Rates: The log disparity metric that has been shown to be effective in measuring fairness in RCTs is ported into the synthetic data domain. We measure the representation of different covariates (protected attributes), both univariate and multivariate, between the synthetic data and the real data from which it was created. The metric is quite powerful, highlighting stratified groups of individuals in a given dataset which are under or over-represented. In our case studies, we found that the unfairness is more representative across cross-section of covariates. For example, in MIMIC we found that not all Whites are highly over-represented but it is the patients who are 81 or more years older males.

The results highlight the effectiveness of the metric to capture not just univariate but also multivariate protected attribute populations. The metric enabled us identify that even when the proportions across subgroups might appear similar and thus, fair, the truth might be revealed after more in-depth exploration. For example, for ATUS, for both gender and age individually, the metric shows the results to be fair. However, when we look at the combination of protected attributes, it becomes apparent that some subgroups are under-represented (female aged 75+) while others are over-represented (males aged 25–34).

Metrics for Temporal Analysis: We introduced a time-series log disparity metric which uses a time-series based metric to measure fairness for temporal synthetic healthcare datasets. The necessity to capture temporal trends of healthcare datasets is highly essential, as health events of an individual are often time-series, each event being a time-point across the life of an individual. As a result, the metric, such as the one defined here, provide a quantifiable measure to identify whether the synthetic data capture this trend or not. From our case studies, we found that temporal datasets are hard for synthetic data generation methods to capture, often leading to unfair representation of certain covariates. The metric is effective in highlighting that while some subgroup populations might be captured adequately, others may be under-represented. This is clearly visible in Figure 6, where Whites are adequately represented while Asians are not.

We identified that synthetic healthcare datasets had problems which did not appear without adequate measurement. Even though they satisfy the overall utility, resemblance and privacy constraints, it appears that they tend to get biased towards certain subgroups to achieve these goals. This is highlighted better using covariate-level fairness analysis, which demonstrate that subgroups are often biased towards one or the other class. Using fairness metrics such as those defined in this article, fairness of synthetic data generation methods can be quantified such that the models can be improved.

Measures of Fairness: The introduction of the fairness in synthetic data problem and the associated metrics in this article are proposed as a starting point for potential future research in this direction. We focused on metrics on the fairness of resemblance between synthetic and real data. We adapted existing methods for measuring similarity of distributions stratified by subgroups to create new fairness metrics. Many other approaches could be used such as differences measured in statistical tests or alternative definitions of fairness. To cater to other definitions of fairness, we invite researchers to develop alternative and potentially even better fairness metrics for synthetic data.

While our work was centered around “fairness and resemblance”, fairness of other aspects of synthetic healthcare data needs to be addressed.

Utility and Fairness: The ability of synthetic health data being useful for any real world application or research is derived from its utility. A synthetic data which has high utility would potentially allow development of models which would give similar results if the models were trained on real data instead. Thus, it is important to realise the utility of any given synthetic dataset. For targeted utility, this would require that the synthetic data generation produces data which have equivalent utility for a fixed analysis tasks for all subgroups of protected attributes. For example, the utility could be the accuracy of a classifier on a testing set. This would involve the measurement of utility across each subgroup, potentially defining the “fairness” of the model for that specific subgroup. Here, existing research in ML-Fairness could be readily adapted. If individual fairness across each subgroup is achieved, we would consider the generation methods to be fair in regards to utility.

Utility and Privacy: While trying to achieve overall privacy preservation, we must also evaluate the individual subgroup-level privacy. We must ensure all subgroups, especially the minority groups, are not exposed to attack or data leakage. Andrew et. al. [16,19] introduced the privacy measurement metrics nearest neighbors Adversarial Accuracy (nnAA) which can potentially be extended to measure privacy across each subgroup. After ensuring subgroup privacy and overall privacy, the resultant dataset could be considered privacy-fair.

Developing Fair Models: Using fairness metrics, the synthetic data bias can be quantified. Using the results from the metrics, new and more fair synthetic data generation models can be developed. Fairness metrics can be incorporated into the design of generation methods much like fairness measures are used in ML-fairness research. Fairness metrics could capture when synthetic data generation may under-represent the prevalence of minority and other subgroups. They could reveal mode collapse or other limitation of GANs mode [24], helping researchers to design and tune more accurate synthetic data generation methods. By ensuring subgroup- level fairness in synthetic data, we can check the proportion (and/or presence) of all subgroups and the quality of their representation in the synthetic data. This helps us to detect any mode collapse that happened during model training and thus, potentially rectify it before the synthetic data is used for real-world application.

Fair Synthetic Data Generation: Given the vast majority of fairness definitions, it is essential to define the generation method and the fairness metric it supports. Thus, this would lead to generation of data which is dependent on the problem and its domain. The use of fairness metrics should be an essential step in the direction of optimizing GANs for more realistic, fair, and private healthcare data.

One possible solution is the inclusion of the fairness metrics as part of the loss function or a regularization penalty, potentially leading to the development of a model that can produce different sub-populations with equal representation. This could be achieved with the introduction of conditional GANs (cGANS) [43] where the under-represented subgroups can be identified and generated in abundance to accommodate their lower representations. The condition would entail the under-represented group, resulting in generation of data specifically for that group such that it has higher proportion.

In synthetic health data generation, we want the synthetic data to closely resemble the real data. In the case studies, the resultant datasets try to preserve the proportions of various subgroups of a given feature. For example, in the ATUS case study, the real data had 13,135 males and 17,391 females while the synthetic data had 12,806 males and 17,194 females. The numbers match closely, which is essential as we want the synthetic data to be an accurate representation version of the real data. However, this might also imply that any bias in the real data due to small sample size is also propagated in the synthetic data. Thus, another possible solution would be to introduce over- and under-sampling of subgroups of a protected attribute as a pre-processing step before feeding it to the generator for training.

Another solution is to use fairness evaluation to determine the under-represented and over-represented classes after training and use it as a feedback loop to the model. This shall involve the downstream classification of synthetically generated healthcare data. The fairness metrics will be evaluated on this dataset, producing the over- and under-representation of certain classes. These insights could be fed back into the synthetic data generator to improve its fairness. Thus, development of synthetic data generation methods must incorporate fairness metrics to generate more robust and representative data.

## 4. Methods

### 4.1. Datasets

For the identification of fairness across covariates, we leveraged three published synthetic datasets from the healthcare domain with both real and synthetic versions.

Impact of Race on 30-day mortality using MIMIC-III Dataset: MIMIC is a large, publicly accessible (with proper approvals) dataset that includes information about patients from Beth Israel Deaconess Medical Center in Boston, Massachusetts. The dataset is available in different versions. MIMIC-II includes ICU stay information for the patients from the year 2001 to 2008. MIMIC-III is an extension to this dataset which includes ICU stay details of over 40,000 patients from the year 2001 to 2012. In [21], the authors used MIMIC-II to create a dataset for understanding the impact of race on 30-day mortality. Andrew et. al. [16] leveraged the idea to create a similar dataset using MIMIC-III as well as created a synthetic version using HealthGAN. We leveraged the real and synthetic versions generated directly for our analysis. The dataset includes three protected attributes: race (White, Black, Asian, Unknown and Other), gender (Male and Female) and age (≤45, 46–65, 66–80, 81+) along with the outcome variable of 30-day mortality: alive (0) and dead (1).

Average Sleep Time of Americans based on ATUS Dataset: American Time Use Survey (ATUS) [14] data consist of survey results and interviews for how subjects spend their time across several activities like sleeping, paid work etc during one day. Dash et. al. [15] derived a time series of data set of minutes of sleep times per hour for each subject and created a synthetic version using HealthGAN. The dataset consists of over 30,000 individuals summarized using covariates of: gender (Male and Female), age-groups (15–24, 25–34, 35–44, 45–54, 55–64, 65–74 and 75+) and day of the week and month collected. For each subject, the number of minutes slept is calculated for each hour for 30 contiguous hours.

Co-occurring Comorbidities in ASD Patients’ Claims Dataset: Autism Spectral Disorder (ASD) claims data include diagnosis of over 280,000 children across 7 Comorbid Medical Conditions (CMCs) recorded for a time-period of 5 years [42]. These 7 CMCs include Auditory disorders (Auditory), Developmental Delays (DD), Gastrointestinal Disorders (Gastro), Immune Disorders (Immune), Psychiatric Disorders (Psychiatric), Seizures (Seizure) and Sleep Disorders (Sleep). The dataset includes children identified with ASD and controls. The data include a combination of protected attributes (2 columns: gender and race). The temporal attributes were created by calculating if each CMC occurred during each 6 month block. The data include a set of 7 time-series, each with 10 time-steps corresponding to 6-month periods. For our analysis, we used the synthetic and real datasets generated based on the prevalence of various diagnosis. Additional synthetic and real datasets exist based on the counts of CMCs occuring at each time set but we exclude this additional for brevity.

### 4.2. Data Generation and Code

The synthetic datasets for the three different datasets were based on HealthGAN, which is a GAN based on Wasserstein GAN with gradient penalty in prior studies. The HealthGAN code is available on the synthetic_data GitHub repository [44]. The synthetic versions of some datasets and the real ATUS dataset are available in another repository. The real MIMIC and ASD datasets are only accessible via permission of the original owners, thus, we cannot release them.

The non-temporal representation metrics with supplementary visualizations and statistical tests were built through R. The time-series log metrics were analysed using Python 3.6 and Python packages numpy and pandas. The code was written in easy to follow Jupyter notebooks which explain each step of the evaluation as well as generate plots using matplotlib and seaborn. The results are then concatenated into dataframes which are colored based on the defined color-scheme. The codes will be published on GitHub upon publication.

### 4.3. Log Disparity Metric and Statistics

ML fairness metrics are developed to evaluate and mitigate potential inequities towards protected subgroups based on predicted outcomes of trained classification models compared with non-protected subgroups. In the context of ML fairness, disparate impact measures the discrimination between the outcome distributions for both unprotected and protected groups [39]:

(8) $$\frac{P(y^{\prime }=1\left|g\left(x\right)=1\right)}{P(y^{\prime }=1\left|g\left(x\right)=0\right)}.$$

Here, *x* is the independent variable or attribute. Both *y* and $y^{\prime }\in \left\{0,1\right\}$ where 1 indicates true and 0 is false. *y* is the true label of a subject, $y^{\prime }=1$ is the label predicted by the classification function, and g(x) is a defined function that determines if a subject is in a subgroup g(x)=1 or not.

We use disparate impact to create a disparity metric for synthetic data by modeling how the real and synthetic data are sampled using an approach first applied to measure disparities in RCTs [34]. We assume that *y* is the outcome of a classification function that indicates if point *x* occurs in the sample of real data, *R*. Similarly, $y^{\prime }$ is the outcome of a classification function that indicates if point *x* occurs in the sample of synthetic data, *S*. In the context of synthetic data, we assume (1) samples from the real distribution and samples generated from the synthetic distribution are independent (i.e., $y⊥y^{\prime }$); and (2) the real data subjects are sampled independent of subgroups (i.e., $y⊥g\left(x\right)$).

It is not obvious how to estimate the probabilities necessary for disparate impact definition (8), $P(y^{\prime }=1\left|g\left(x\right)=1\right)$ and $P(y^{\prime }=1\left|g\left(x\right)=0\right)$, from the real and synthetic data. Thus, we show that when applied to the synthetic data generation, the ML metric disparate impact reduces to an intuitive quantity based on the ratio of odds of generating a subject from the protected subgroup in the synthetic data and the odds of sampling a subject from the subgroup in the real distribution. This transformation is essential to estimate probabilities in the definition (8) from the real and synthetic data. The necessary probabilities, P(g(x)=1|y=1)=P(g(x)=1|x∈R) and $P(g\left(x\right)=1\left|y^{\prime }=1\right)=P(g\left(x\right)=1\left|x\in S\right)$, are easily estimated from the actual real and synthetic data.

**Theorem** **1.** *Synthetic Data Equity Version of ML-Fairness Disparate Impact Metric Based on the assumptions described above, the Disparate Impact metric applied to synthetic data is equivalent to the ratio of odds of subjects of the protected group in the synthetic data to the ratio of the odds of subjects in the real data. Recall that $\mathrm{odds}\left(p\right)=\frac{p}{\left(1-p\right)}.$*

**Proof.**  $$\begin{array}{ccc} \frac{P(y^{\prime }=1\left|g\left(x\right)=1\right)}{P(y^{\prime }=1\left|g\left(x\right)=0\right)}\; & =\frac{\left(P\left(y^{\prime }=1,g\left(x\right)=1\right)\right)/\left(P\left(g\left(x\right)=1\right)\right)}{\left(P\left(y^{\prime }=1,g\left(x\right)=0\right)\right)/\left(P\left(g\left(x\right)=0\right)\right)}\\ \; & =\frac{(P\left(g\left(x\right)=1\left|y^{\prime }=1\right)P\left(y^{\prime }=1\right)\right)/\left(P\left(g\left(x\right)=1\right)\right)}{(P\left(g\left(x\right)=0\left|y^{\prime }=1\right)P\left(y^{\prime }=1\right)\right)/\left(P\left(g\left(x\right)=0\right)\right)}\\ \; & =\frac{P(g\left(x\right)=1\left|y^{\prime }=1\right)P(g\left(x\right)=0\left|y=1\right)}{P(g\left(x\right)=0\left|y^{\prime }=1\right)P(g\left(x\right)=1\left|y=1\right)} & sincey⊥g(x)\\ \; & =\frac{P(g\left(x\right)=1\left|y^{\prime }=1\right)(1-P\left(g\left(x\right)=1\left|y=1\right)\right)}{(1-P\left(g\left(x\right)=1\left|y^{\prime }=1\right)\right)P(g\left(x\right)=1\left|y=1\right)}\\ \; & =\frac{P(g\left(x\right)=1\left|y^{\prime }=1\right)}{1-P(g\left(x\right)=1\left|y^{\prime }=1\right)}/\frac{P(g(x)=1|y=1)}{1-P(g(x)=1|y=1)}\\ \; & =\frac{\mathrm{odds}(g\left(x\right)=1\left|y^{\prime }=1\right)}{\mathrm{odds}(g\left(x\right)=1\left|y=1\right)}\\ \; & =\frac{\mathrm{odds}(g\left(x\right)=1\left|x\in S\right)}{\mathrm{odds}(g\left(x\right)=1\left|x\in R\right)} \end{array}$$

□

Taking a natural logarithm improves ease of understanding of the equity measurements by making under-represented and over-represented metric symmetric. For example, if gender = male or female, then the log disparity of the male subgroup will be the negative of the log disparity of the female subgroup. Thus, the log disparity metric is defined as below.

(9) $$\log\left(\frac{\mathrm{odds}(g(x)=1|x\in R)}{\mathrm{odds}(g(x)=1|x\in S)}\right).$$

If we consider the synthetic and real data to be generated by sampling, it is possible that any differences observed may be by chance. Thus, we develop a statistical test with the null hypothesis P(g(x)=1|x∈R)=P(g(x)=1|x∈S). Let $p_{S}=P\left(g\left(x\right)=1|x\in S\right)$ with size $n_{S}$ for the subgroup in the synthetic data and $p_{R}=P\left(g\left(x\right)=1|x\in R\right)$ with size $n_{R}$ for the subgroup in the real data. *p* is the overall proportion. Then a two-proportion z-test Equation (10) is applied.

(10) $$z=\frac{p_{S}-p_{R}}{\sqrt{\frac{p(1-p)}{n_{S}}+\frac{p(1-p)}{n_{R}}}}.$$

The z-test is applied when the sample size is large enough; otherwise, more accurate test for small samples such as the Fisher Exact probability test is used. We note that this t-statistic could also provide an excellent basis for another novel fairness metric for synthetic data as well.

We calculate the log disparity metrics and statistical significance for all possible subgroups defined by the protected attributes by changing g(x). Here, we assume that the protected attributes are all categorical. Since multiple subgroups are compared, we use the Benjamini–Hochberg procedure to adjust p-values through controlling the false discovery rate. If the adjusted *p*-value is greater than or equal to the significance level (0.05), no statistically significant difference between real and synthetic subgroup representation exist. Then, we will apply the metric such as log disparity for further evaluation.

### 4.4. Time-Series Metrics

In this article we introduce a time-series metric based log disparity metric. We use Pearson’s Correlation Coefficient (PCC) and Directional Symmetry (DS) for res() from existing time-series literature [45,46] as example metrics in this article but the concept can be extended to other metrics as well. If ${\left\{a^{k}\right\}}_{k=1}^{N}$ and ${\left\{b^{k}\right\}}_{k=1}^{N}$ represent the real and synthetic time-series for a given subgroup of a protected variable, respectively, then, we define our two time-series metrics as:

Pearson’s Correlation Coefficient (PCC): PCC captures the linear correlation between the real and synthetic data, measuring the relationship between the two time-series. Its value ranges between −1 and 1 where −1 would imply complete inverse-correlation while 1 implies complete correlation between the two series. However, as we would use this metric with natural log, we convert the values to 0 and 1. The modified formula is as follows:

(11) $$\mathrm{PCC}=\frac{\frac{\mathrm{cov}(a,\;b)}{\sigma_{a}\sigma_{b}}+1}{2}.$$

Directional Symmetry (DS): DS is a percentage measure to understand the ability of synthetic data to capture the direction of the real data trend in the given subgroup.

(12) $$\mathrm{DS}=\frac{100}{N-1}\sum_{k=1}^{N-1}d_{k},\;\mathrm{where}\;d_{k}=\left\{\begin{array}{cc} 1, & \mathrm{if}\left(a^{k+1}-a^{k}\right)\left(b^{k+1}-b^{k}\right)\geq 0\\ 0, & \mathrm{otherwise} \end{array}\right..$$

PCC discounts for distortions due to changes in location or/and scale of the two series while DS focuses on concordance of the upward and downward variations, making it a bit more forgiving. We specifically chose to select these two metrics for resemblance as they cover two different aspects. We leverage these metrics in disparate impact for time-series formula defined above and evaluate the temporal fairness of protected attributes. This can be extended to myriad other metrics that exist in the time-series literature but has been left as a potential future work. The best statistical test for statistical significance of time series metrics is also left as future work. We use the thresholds for over and under representations defined in the previous sections.

## Data Availability

The data presented in this study for MIMIC-III is derived from the data available in PhysioNet at https://doi.org/10.13026/C2XW26 (accessed on 24 June 2021) which can be obtained with aproval from PhysioNet. The synthetic version of the MIMIC-III derived dataset is available at https://github.rpi.edu/RensselaerIDEA/SyntheticDataFairness/tree/master/data/Mimic (accessed on 24 June 2021). The datasets derived from the American Time Use Survey (ATUS) including both real and synthetic are available at https://github.rpi.edu/RensselaerIDEA/SyntheticDataFairness/tree/master/data/Atus (accessed on 24 June 2021). Restrictions apply to the availability of the real Autism Spectrum Disorder (ASD). The ASD dataset was obtained from OptumLabs^®^ Data Warehouse (OLDW) and cannot be accessed outside their secure environment, thus, no link for the same is available.
